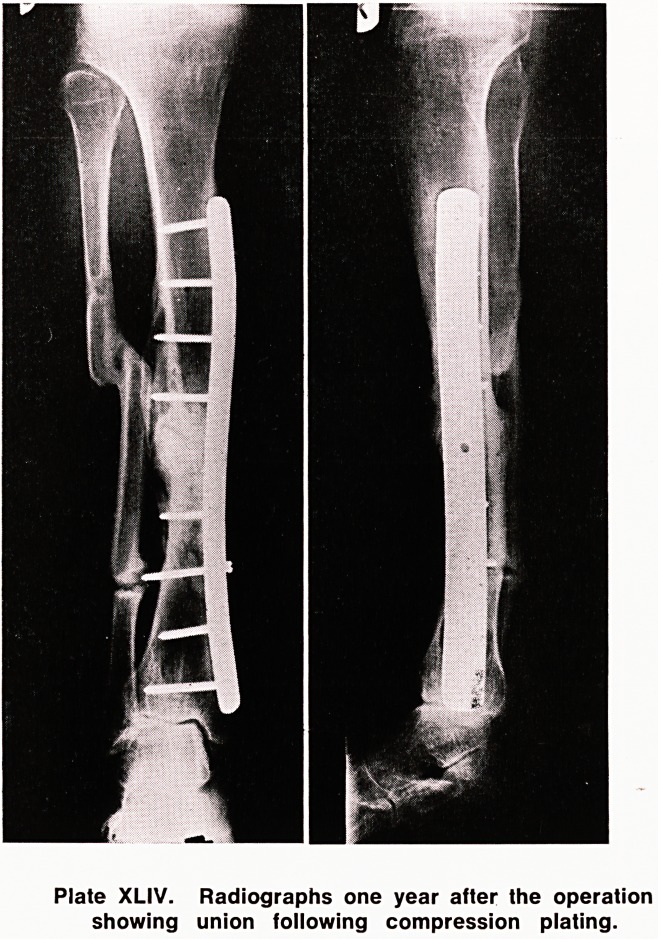# Compression Plating for Non-Union of the Tibia

**Published:** 1971-10

**Authors:** E. Michelinakis

**Affiliations:** Orthopaedic Registrar, Southmead Hospital, Bristol


### Compression Plating for Non-Union of the Tibia

**E. Michelinakis, M.D.**

Orthopaedic Registrar, Southmead Hospital, Bristol

Compression plating as a method of treatment for the fractures of the long bones has gained increased popularity in orthopaedics during the last decade. The reasons are twofold: firstly, the surgeon's desire for a rigid fixation which will facilitate rapid and certain union, secondly, the patient's preference for early ambulation without the necessity for plaster of paris. In 1932, Albert Key was the first to use compression for arthrodesis of the knee joint in which, after excision of the joint, the cut bone surfaces were compressed against each other. In 1948, John Charnley studied the influence of compression on bone union. In the Bristol Royal Infirmary, intensive research started five years ago on compression plating. One hundred and fifty-four fractures of the tibia have undergone this procedure and no cases of non-union have been found.

The most obvious effect of compression in the fracture site is the impaction of the bony trabaculae until they interdigitate, and radiologically the fracture line is no longer apparent. Consequently, the space to be filled with new bone is small and in the absence of rotatory and shearing movements, the produced callus is used to the best mechanical advantage. Mobilization of the affected limb in the post-operative period reduces joint stiffness, muscle wasting and subsequently shortens the convalescence.

Fractures of the shaft of the tibia have a greater tendency to delayed union or non-union than those of any other long bone. The following case clearly illustrates this problem and the effect of the compression plating on an ununited fracture of the tibia where every other method of treatment had failed.

**CASE REPORT**

A 20 year old male patient was admitted to one of the Liverpool Group Hospitals in August 1965 following a motor-cycle accident, with a comminuted fracture of the right tibia (Plate XXXIX).

The tibia was immobilized in plaster of paris for nine months, but in spite of this unusually long period in plaster the fracture did not unite (Plate XL).

**Plate XXXIX. Radiographs of a grossly comminuted fracture of the tibia.**

**Plate XL. The fracture has not united, in spite of 9 months in plaster of paris.**

Internal fixation was carried out using a simple plate with four screws. Bone grafts were taken from the left iliac crest and tied around the fracture site with wire, immobilization in plaster of paris being continued (Plate XLI).

**Plate XLI. Simple plating was performed. The wires held the bone-grafts on the fracture site.**

He was immobilized thus for a further six months, but the fracture still failed to unite. The plate was subsequently removed eleven months after the first operation, and bone grafts taken from the right iliac crest were again placed around the fracture (Plate XLII).

**Plate XLII. The plate was removed eleven months from the operation and a second bone grafting was performed.**

In December 1967, seven months after the second operation and two years and four months after the initial injury, the fracture was still mobile. It was explained to the patient that this had to be treated as an ununited fracture and he was fitted with full length caliper and discharged from the clinic to attend periodically for renewal of his caliper.

In March 1970 he attended out-patients with another problem. He wished to be married and wondered whether anything could be done to unite his tibia. On examination, the fracture was mobile and painless. There were two large healed scars present on the skin, and the neighbouring joints had a good range of movements. The x-rays (Plate XLIII) showed the gap between the bone ends, sclerosis, and moulding of the fracture surfaces.

**Plate XLIII. The radiograph shows the fracture as it was 5 years after the initial injury. Pseudarthrosis was present.**

A compression plate was inserted with compression forces of about 25 lb. The leg was left out of plaster post-operatively and exercises were commenced on the following day. Five weeks after the operation he was allowed to bear weight. An x-ray (Plate XLIV) taken one year after the operation showed that the gap was bridged with continuation of the bony trabeculae. The sclerotic bone has revitalized.

**Plate XLIV. Radiographs one year after the operation showing union following compression plating.**

He has since married.

**SUMMARY**

A 25 year old patient, with an ununited fracture of the tibia has been successfully treated with compression plating five years after the initial injury. Previous measures included prolonged immobilization in plaster of paris, plate fixation, bone grafting and the application of a long-leg caliper.

---

I should like to thank Mr. Keith Lucas for his expert advice in dealing with this case and Mr. Harry Griffiths for his criticism. I am indebted to the Department of Medical Photography, Frenchay Hospital for the photographic work.

**REFERENCES**

1. **Charnley, J. C.** (1948): Positive Pressure in Arthrodesis of the Knee Joint. Journal of Bone and Joint Surgery. **30-13:** 478-486.

2. **Key, J. A.** (1932): Positive Pressure in Arthrodesis for Tuberculosis of Knee Joint. Southern Medical Journal. **25:** 909-915.

3. **Lucas, H. K.** (1971): Personal Communication.

## Figures and Tables

**Plate XXXIX. f1:**
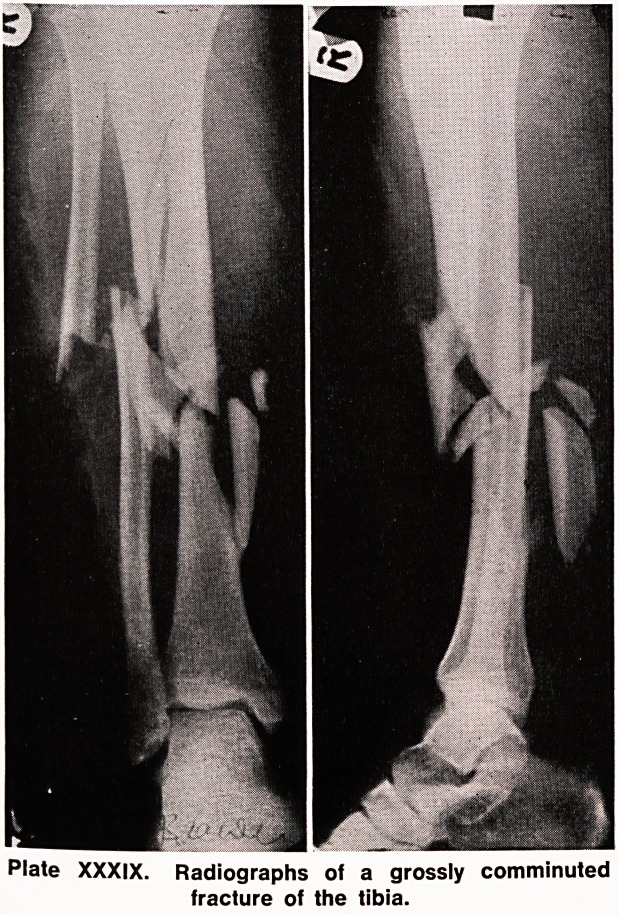


**Plate XL. f2:**
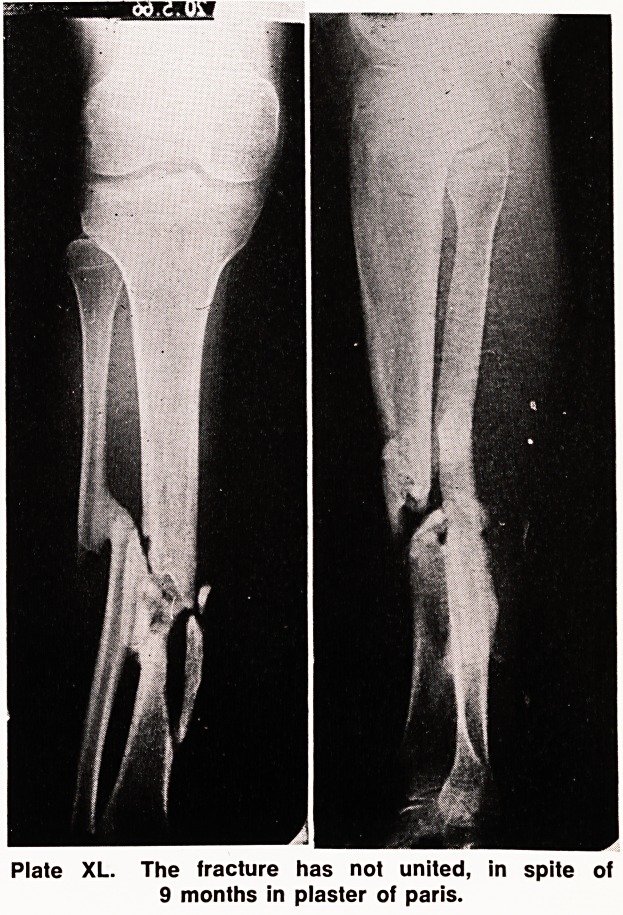


**Plate XLI. f3:**
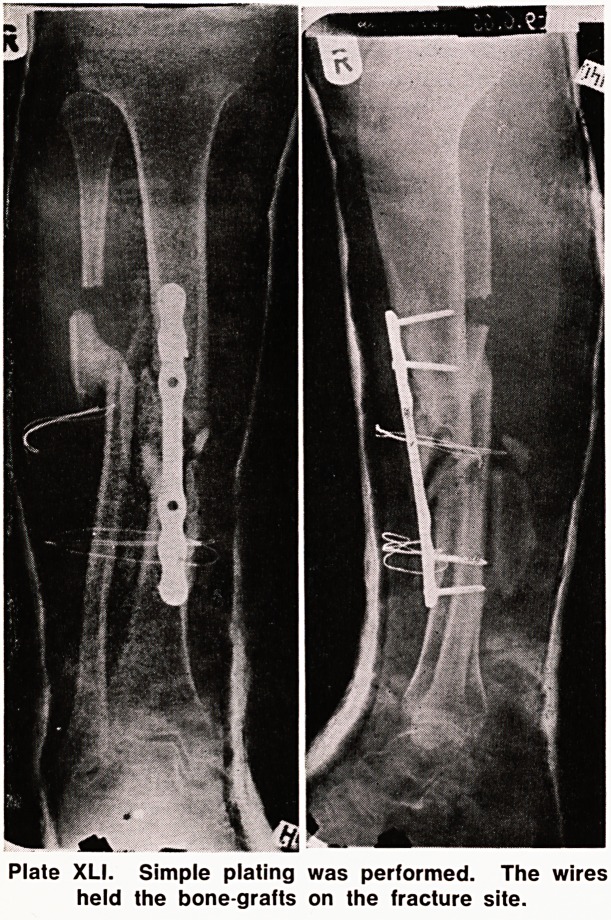


**Plate XLII. f4:**
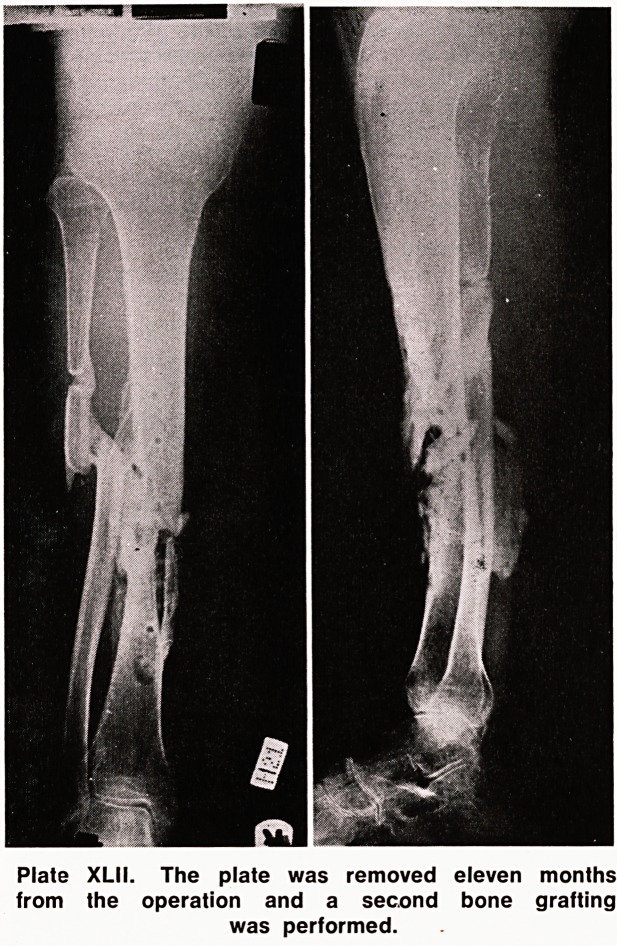


**Plate XLIII. f5:**
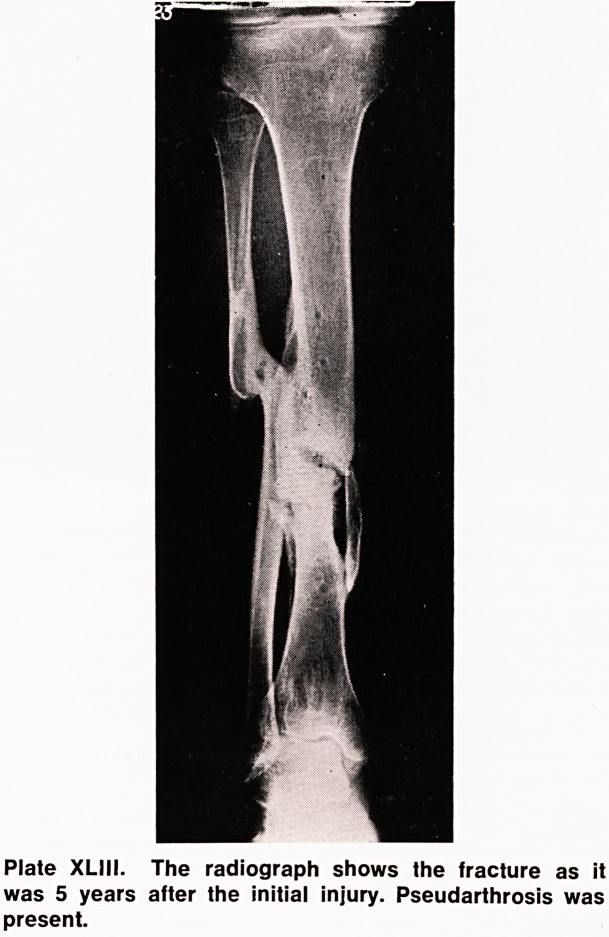


**Plate XLIV. f6:**